# Dorsal Root Ganglion-Targeted DNA Origami Delivery of IL1RN for Skeletal Growth and Repair

**DOI:** 10.3390/pharmaceutics18070898

**Published:** 2026-07-22

**Authors:** Yumiao Jiang, Xinyi Gu, Zenglin Yin, Shen Wang, Jin Deng, Shuhang Guo, Xiaofeng Yin

**Affiliations:** 1 Department of Trauma and Orthopedics, Peking University People’s Hospital, Beijing 100044, China; 2 Center for Plastic & Reconstructive Surgery, Department of Plastic & Reconstructive Surgery, Zhejiang Provincial People’s Hospital (Affiliated People’s Hospital), Hangzhou Medical College, Hangzhou 310014, China; 3 Beijing Institute of Pharmacology and Toxicology, Beijing 100850, China

**Keywords:** IL1RN, DNA origami, sensory nerves, dorsal root ganglion, bone homeostasis, bone repair, targeted drug, cartilage, neuro-regulation, age-related bone loss

## Abstract

**Background/Objectives:** Sensory nerves, as essential peripheral nerves, innervate bone and release various neuroactive substances—including neurotransmitters, neuropeptides, and neurocrine factors—that participate in bone growth, remodeling, and metabolism. Interleukin-1 receptor antagonist (IL1RN), an endogenous anti-inflammatory mediator, is a key regulatory molecule in the pathogenesis of inflammatory diseases such as osteoarthritis and rheumatoid arthritis. However, its role as a sensory neurocrine factor in the regulation of bone tissue has rarely been investigated. This study aimed to explore the regulatory effects of sensory nerve–derived IL1RN on bone tissue. **Methods**: A dorsal root ganglion (DRG)-targeted delivery system was developed using DNA origami technology to load IL1RN protein or IL1RN-targeting siRNA and was functionalized with a DRG-homing peptide. Bone defect and age-related bone loss models were established in C57BL/6 mice to preliminarily investigate the regulatory role of IL1RN secreted from sensory nerve endings in bone tissue. **Results:** IL1RN suppressed bone resorption and promoted new bone formation at defect sites. In the age-related bone loss model, IL1RN preserved the integrity of the growth plate. These findings indicate that sensory nerve–derived IL1RN may participate in the regulation of bone repair and skeletal homeostasis. **Conclusions:** IL1RN may serve as a potential therapeutic target for DRG-mediated regulation of bone repair. These findings suggest that DRG-targeted modulation of IL1RN may represent a potential approach for investigating and regulating sensory nerve–associated bone repair.

## 1. Introduction

Bone is a highly dynamic mineralized connective tissue that maintains its homeostasis through continuous renewal and remodeling. This process is achieved through the coordinated action of a transient anatomical structure called the basic multicellular unit (BMU), which consists of osteoblasts, osteocytes, bone-lining cells, and osteoclasts [1]. Physiological bone remodeling is essential for skeletal repair and the maintenance of mechanical strength. An imbalance between osteoblast-mediated bone formation and osteoclast-mediated bone resorption leads to pathological alterations. For instance, excessive activation of osteoclasts results in progressive bone loss and osteoporosis, whereas insufficient resorption gives rise to osteopetrosis. This remodeling process is regulated by the “peripheral nerve-bone” network [2,3,4,5,6].

Primary sensory nerves, originating from the dorsal root ganglion (DRG), represent major peripheral nerves innervating the skeleton. Their fibers widely cover the bone surface and partially extend into mineralized bone and the bone marrow cavity. In particular, at sites of active bone remodeling, such as fracture healing regions and the epiphyseal growth plate, sensory fibers undergo re-arrangement and re-innervation to achieve specific localized enrichment. This spatial heterogeneity suggests that sensory nerves play a pivotal role in bone regeneration, bone mass accumulation, and metabolic regulation [7,8,9].

In skeletal diseases, accumulating evidence indicates that sensory nerves not only perform their fundamental function of nerve fibers, such as transmitting external and internal stimuli to the central nervous system via neural electrical signals, but also modulate bone homeostasis through a unique neuro–bone axis by releasing various neuroactive substances [10,11]. For example, a co-culture study using in vitro DRG-derived neurons and MC3T3-E1 cell lines to explore the communication mechanism between sensory nerves and osteoblasts revealed that sensory neuropeptide substance P (SP) positively regulates bone mass [12]. With further investigation, calcitonin gene-related peptide (CGRP), which is a peptide substance synthesized, stored, and released predominantly by sensory neurons, has been demonstrated to promote bone formation and inhibit bone resorption. Moreover, CGRP facilitates angiogenesis at bone injury sites by upregulating vascular endothelial growth factor (VEGF) and hypoxia-inducible factor (HIF), while also modulating the immune microenvironment of bone repair through enhancing the polarization of macrophages from the M0 to M2 phenotype [13,14,15]. Another study aimed at investigating the mechanism of silicified collagen scaffolds on sensory nerve–mediated bone defect healing identified semaphorin 3A (Sema3A) as a key factor that promotes in situ bone regeneration [16]. Collectively, these findings reveal sensory nerves as regulatory hubs of bone homeostasis and remodeling, playing critical roles in bone repair.

To further investigate the impact of sensory nerves and their neurocrine factors on bone, we established a rat tibial fracture model in preliminary experiments and performed transcriptome sequencing of both fracture sites and DRG tissues at 0, 3, 7, 14, and 28 days. The results of differential expression gene analysis showed that the upregulation of the interleukin-1 receptor antagonist gene (IL1RN) at 28 days caught our attention. IL1RN encodes the endogenous cytokine interleukin-1 receptor antagonist (IL1Ra), a crucial inflammatory mediator that competitively inhibits the binding of IL-1 to its receptor, interleukin-1 receptor type I (IL1R1). By blocking the formation of the IL1R1/IL-1 receptor accessory protein complex and subsequent MyD88-dependent signaling, IL1RN suppresses IL-1-mediated inflammatory responses [17,18]. Although IL1RN has been extensively studied as a key regulatory factor in inflammatory diseases such as rheumatoid arthritis and osteoarthritis, its role in bone homeostasis and remodeling remains unclear [19,20]. In this study, we developed a dorsal root ganglion-targeted DNA origami delivery system for IL1RN modulation and evaluated its targeting specificity, neurotoxicity, and effects on skeletal growth and repair in mouse models.

## 2. Materials and Methods

### 2.1. Experimental Animals

C57BL/6 mice (6–8 weeks old, weighing 22–25 g) and aged male C57BL/6 mice (12 months old, weighing 28–35 g) were obtained from Beijing VTLH Laboratory Animal Co., Ltd. (Beijing, China). Mice were housed in the animal facility of Peking University People’s Hospital under controlled conditions: room temperature maintained at 20–22 °C, ad libitum access to food and water, and a 12-h light-dark cycle. Mice were housed in groups of three per cage. This study was approved by the Animal Ethics Review Committee of Peking University People’s Hospital and complied with ethical regulations for laboratory animals (Approval No: 2020PHE089).

### 2.2. Design and Fabrication of DNA Origami Drug Delivery Systems

Triangular DNA origami structures were self-assembled according to established protocols [21]. M13mp18 scaffold DNA was purchased from New England Biolabs (Cat. No. N4040S; Ipswich, MA, USA). Staple strands, siRNA-capture strands, peptide-capture strands, biotinylated oligonucleotides, 5′-Cy5-modified oligonucleotides, and other functionalized DNA strands were synthesized by Sangon Biotech Co., Ltd. (Shanghai, China). Detailed sequences are provided in Supplementary Files S1 and S2.

Triangular DNA origami nanostructures (DONs) were assembled using M13mp18 scaffold DNA and the corresponding staple strands. Briefly, M13mp18 scaffold DNA was mixed with staple strands and functionalized oligonucleotides at a scaffold-to-staple molar ratio of 1:5 in 1× TAE-Mg^2+^ buffer. The buffer contained 40 mM Tris, 20 mM acetic acid, 2 mM EDTA, and 12.5 mM magnesium acetate, pH 8.0. The mixture was subjected to programmed thermal annealing in a PCR thermocycler by gradually cooling from 95 °C to 25 °C over 2.5 h. After annealing, the assembled functionalized DONs were purified using Amicon Ultra-0.5 mL 100 kDa centrifugal filters (Millipore Corporation, Bedford, MA, USA) to remove excess staple strands. The purified functionalized DONs were characterized by atomic force microscopy to confirm the main nanostructure morphology and were stored at 4 °C until further use.

To confer dorsal root ganglion (DRG)-targeting capability, a C-terminal cysteine-modified DRG-homing peptide (sequence: SPGARAF-Cys; Sangon Biotech Co., Ltd., Shanghai, China) was conjugated to NH_2_-modified linker DNA (sequence: 5′-NH_2_-C6-ACACACACACACACACACA-3′) through Sulfo-SMCC [sulfosuccinimidyl 4-(N-maleimidomethyl)cyclohexane-1-carboxylate]-mediated coupling. Briefly, NH_2_-modified linker DNA was reacted with Sulfo-SMCC at a molar ratio of 1:10 at room temperature for 10 h. Excess Sulfo-SMCC was removed by repeated ultrafiltration using 3 kDa ultrafiltration units. The resulting maleimide-functionalized DNA intermediate was then mixed with SPGARAF-Cys at a molar ratio of 1:10 and reacted at room temperature for 6 h. Excess SPGARAF-Cys was removed by 3 kDa ultrafiltration to obtain purified SPGARAF-DNA.

The purified functionalized DONs were mixed with SPGARAF-DNA at a DON-to-SPGARAF-DNA molar ratio of 1:5n, where n represents the number of designed SPGARAF-loading sites on the DON surface. The mixture was subjected to programmed annealing from 45 °C to 25 °C over 1 h, allowing SPGARAF-DNA to hybridize with the complementary peptide-capture strands on the DON surface. The resulting formulation was designated SPGARAF-DON.

For the IL1RN protein-loaded formulation, IL1RN protein was loaded onto DONs through the streptavidin–biotin interaction. In the designed triangular DON, six IL1RN protein-loading sites were arranged on each edge, corresponding to a total of 18 designed IL1RN protein-loading sites per DON. IL1RN protein was first biotinylated using Sulfo-NHS-biotin. SPGARAF-DON was first incubated with streptavidin at a DON-to-streptavidin molar ratio of 1:5n for 30 min, where n represents the number of designed IL1RN protein-loading sites. After removal of excess streptavidin by ultrafiltration, the resulting streptavidin-functionalized SPGARAF-DON was incubated with biotinylated IL1RN protein at a DON-to-biotinylated IL1RN molar ratio of 1:10n for 30 min. Unbound biotinylated IL1RN protein was removed by ultrafiltration to obtain IL1RN-SA-SPGARAF-DON.

For the IL1RN knockdown formulation, IL1RN-targeting siRNA fragments (Sangon Biotech Co., Ltd., Shanghai, China) were synthesized with 2′-O-methyl (2′-OMe) modifications to enhance stability. A 3′ poly-A-containing overhang was introduced into the siRNA construct to enable hybridization with the poly-T-containing siRNA-capture strands predesigned on the DON surface. SPGARAF-DON was mixed with DNA-overhang-modified IL1RN-targeting siRNA at a DON-to-siRNA molar ratio of 1:2n, where n represents the number of designed siRNA-loading sites on the DON surface. The mixture was incubated in 1× TAE-Mg^2+^ buffer at 37 °C and 1000 rpm for 30 min to obtain siRNA-SPGARAF-DON.

Two Cy5-modified oligodeoxynucleotides were incorporated on each edge of the triangular DON to visualize tissue uptake and distribution.

**Figure 1:**
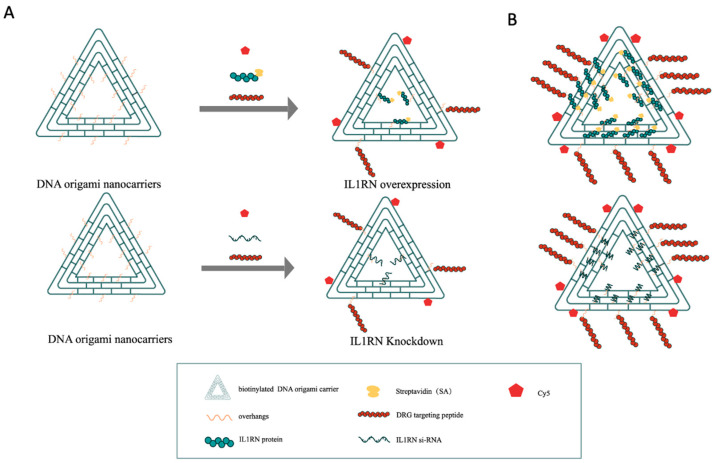
Schematic diagram of IL1RN knockdown and overexpression drugs. (**A**) Flowchart of drug self-assembly with IL1RN overexpression and knockdown. (**B**) Drug design schematic.

The DON was designed as a triangular nanostructure. On each edge, three DRG-homing peptides were assembled at positions 04, 20, and 49 with approximately 20-nm spacing. Six IL1RN protein-loading sites or IL1RN siRNA-loading sites were arranged on each edge at positions 05, 06, 21, 42, 43, and 57, also with approximately 20-nm spacing. Two Cy5 fluorophores were assembled on each edge at positions 30 and 61 for fluorescence tracing (Figure 1).

The schematic illustration was generated using Adobe Illustrator (version 29.7; Adobe Inc., San Jose, CA, USA).

### 2.3. Characterization of the Drug Delivery System

After assembly of the unloaded DON scaffold, the IL1RN-overexpression formulation, and the IL1RN-knockdown formulation, freshly cleaved mica substrates were prepared by attaching mica sheets to silicon wafers with double-sided adhesive and removing the upper mica layer with adhesive tape. A 5 μL aliquot of each sample solution was deposited onto the freshly cleaved mica surface and incubated for 3 min. The substrate was then rinsed with distilled water to remove unadsorbed samples and dried under a stream of high-purity nitrogen gas. Atomic force microscopy (AFM) imaging was performed using a RTESP-300 probe (Bruker, Camarillo, CA, USA) in Tapping Mode with a drive amplitude of 45 mV to confirm the morphology of the DNA origami–based constructs.

To evaluate the stepwise assembly of the DNA origami nanostructure (DON)-based formulations, 1% agarose gels were prepared in 1× TAE-Mg^2+^ buffer and electrophoresed at 90 V for 90 min. After electrophoresis, DNA bands were visualized under UV light using a gel imaging system (Tanon Science & Technology Co., Ltd., Shanghai, China).

### 2.4. In Vivo Evaluation of Drug Targeting Efficiency

Mice were subjected to intrathecal injection using a 27-G needle with the bevel facing the head. The needle was inserted at a 70° angle between the L5–L6 vertebrae. Upon contacting bone, the angle was adjusted to 30° and advanced into the intervertebral space. Correct entry was indicated by reflexive twitching of the tail or hind limbs, after which 20 μL of the drug was injected into the subarachnoid cavity.

After 4 weeks of intrathecal injection, the cervical, thoracic, and lumbar DRGs, together with the lumbar sympathetic ganglia (SG), were collected, fixed in 4% paraformaldehyde, cryosectioned, and subjected to immunofluorescence staining of neurons. The primary antibody was rabbit anti-NeuN (Abcam, Cambridge, UK; Cat. No. ab177487, 1:200), and the secondary antibody was goat anti-rabbit IgG H&L (Alexa Fluor^®^ 488; Abcam, ab150077, 1:400). Cy5-positive neurons were visualized by a fluorescence microscope (Eclipse TS100, Nikon, Tokyo, Japan). The proportion of Cy5-positive neurons relative to all neurons was quantified using ImageJ software (version 2.16.0).

### 2.5. Neurotoxicity Assessment

Brain tissues were collected 2 and 4 weeks after intrathecal injection of the IL1RN-overexpression formulation and were subjected to hematoxylin and eosin (H&E) staining, Nissl staining, and Iba1 immunofluorescence staining. The primary antibody was rabbit anti-Iba1 (Abcam, ab178846, 1:200), and the secondary antibody was goat anti-rabbit IgG (Alexa Fluor^®^ 488; Abcam, ab150077, 1:400).

### 2.6. Western Blotting

After 4 weeks of intrathecal drug administration in mice, dorsal root ganglia (DRG) were harvested. Western blotting was used to assess IL1RN-encoded protein expression levels in DRG tissue from the IL1RN overexpression and knockdown groups.

β-tubulin (Proteintech, Wuhan, China; Cat. No. 10094-1-AP, 1:1000) was used as the loading control, and IL1Ra (Abcam, Cambridge, UK; Cat. No. ab124962, 1:1000) was used as the target protein. The secondary antibody was goat anti-rabbit IgG-HRP (Servicebio, Wuhan, China; Cat. No. GB23303, 1:3000).

### 2.7. Establishment of the Bone Defect Model

C57BL/6 mice (6–8 weeks old, weighing 22–25 g) were anesthetized by isoflurane inhalation using an anesthesia machine, with an induction flow rate of 4–5% and a maintenance flow rate of 1.5–2.5%. Before surgery, hair on the hindlimb was removed, and the surgical site was disinfected with povidone-iodine. A longitudinal skin incision was made along the femoral axis on the lateral thigh. The fascia and muscle were bluntly dissected to expose the femoral surface. A unicortical defect (1 × 2 mm) was created on one side of the femoral cortex using a handheld microdrill without penetrating the contralateral cortex. The surgical site was irrigated with saline, and the skin incision was closed with interrupted sutures. After surgery, the mice were allowed free movement. Throughout the procedure, body temperature was maintained at 37 °C using a heating pad to prevent hypothermia under anesthesia.

### 2.8. Histology

At 2 and 4 weeks post-treatment, femurs were harvested and fixed in 4% paraformaldehyde, decalcified in 10% EDTA, dehydrated through a graded ethanol series, and embedded in paraffin. Sections were cut at a thickness of 10 μm and stained with hematoxylin and eosin. For cartilage evaluation, bone tissue sections prepared as described above were stained with Safranin O and Fast Green. The proportion of Safranin O-positive area relative to the total area was quantified using ImageJ software.

### 2.9. Statistical Analysis

All data were analyzed using SPSS 24.0 statistical software. Quantitative data are presented as the mean ± standard deviation (SD). Comparisons between two groups were performed using Student’s *t*-test. For comparisons among three or more groups, one-way analysis of variance (ANOVA) followed by appropriate post hoc testing was used. A value of *p* < 0.05 was considered statistically significant.

## 3. Results

### 3.1. Design and Construction of IL1RN-Targeted DNA Origami Drugs

**Figure 2:**
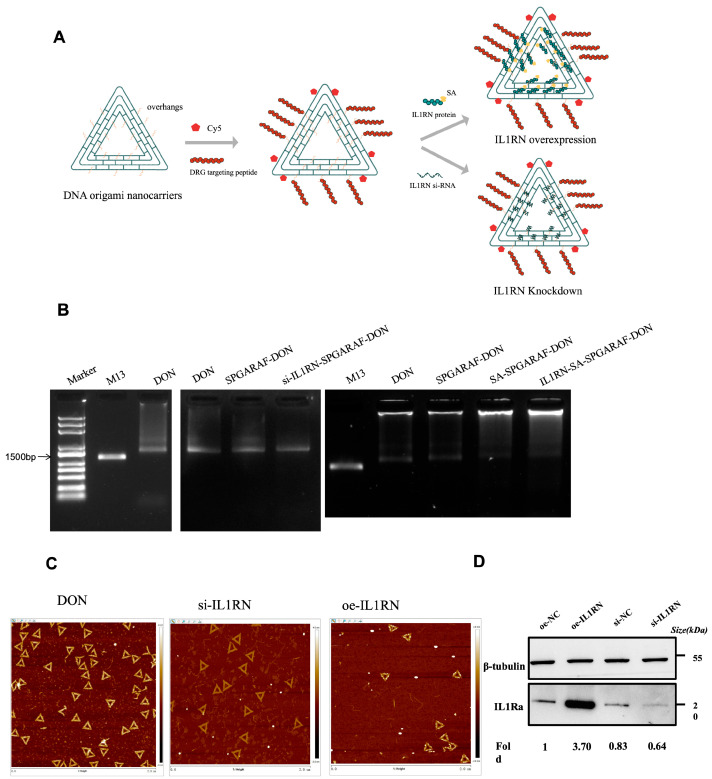
Design and characterization of IL1RN-targeted DNA origami drugs. (**A**) Schematic diagram showing the synthesis process of IL1RN overexpression and knockdown constructs. (**B**) Agarose gel electrophoresis was performed to evaluate the stepwise assembly and functional modification of DNA origami nanostructure (DON)-based formulations. (**C**) Representative AFM images of the unloaded DON scaffold, IL1RN-knockdown formulation, and IL1RN-overexpression formulation. (**D**) Western blot analysis of IL1RN protein expression in DRG 4 weeks after intrathecal drug administration.

In this study, DNA origami drugs were constructed to achieve IL1RN overexpression and knockdown specifically targeting the DRG. Nucleotide strands with predefined sequences were self-assembled into a three-dimensional triangular structure with defined thickness. DRG-homing peptides were conjugated to endow the constructs with DRG-targeting capacity, and Cy5 fluorophores were incorporated to enable visualization of drug uptake within tissues. In the overexpression drug, IL1RN-encoded proteins were assembled onto the DNA origami structure via the strong affinity between streptavidin and biotin. In the knockdown construct, IL1RN-targeting siRNA fragments were synthesized with 2′-O-methyl (2′-OMe) modifications to enhance stability. A 3′ poly-A-containing overhang was introduced into the siRNA construct to hybridize with complementary siRNA-capture strands on the triangular DNA scaffold (Figure 2A). Agarose gel electrophoresis was used to assess the assembly of the DON-based formulations (Figure 2B), suggesting the stepwise construction of the targeted IL1RN protein-delivery and siRNA-delivery formulations. The morphology of the DNA origami structures was further characterized by atomic force microscopy (AFM), which showed intact triangular nanostructures in the unloaded DON scaffold, the IL1RN-knockdown formulation, and the IL1RN-overexpression formulation (Figure 2C). After 4 weeks of intrathecal administration in mice, DRG tissues from cervical, thoracic, and lumbar segments were collected, and Western blot analysis verified local modulation of IL1RN protein expression in the DRG. The results demonstrated that the overexpression drug successfully increased IL1RN expression, whereas the knockdown drug reduced it (Figure 2D).

### 3.2. Tissue Specificity and Low Neurotoxicity of Intrathecally Delivered DNA Origami Drugs

**Figure 3:**
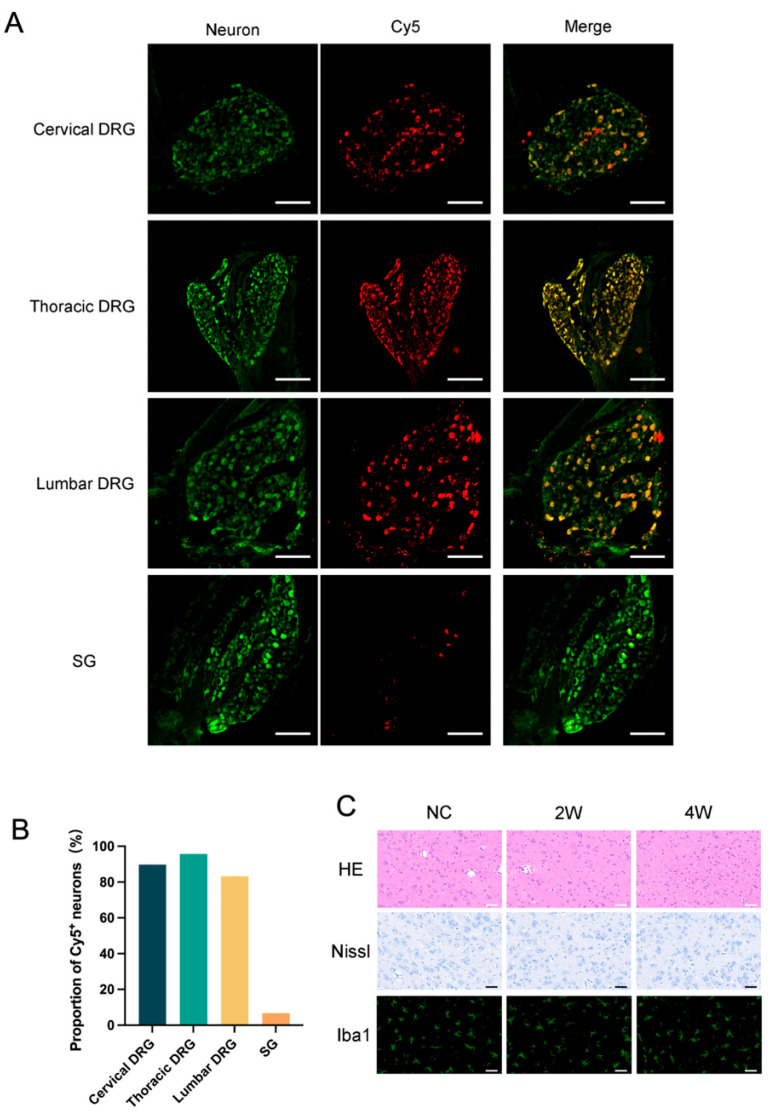
Tissue specificity and neurotoxicity assessment of DNA origami drugs. (**A**) Immunofluorescence staining of DRGs and sympathetic ganglia (SG) of mice following intrathecal drug injection. Green indicates all neurons; red indicates Cy5^+^ neurons. *n* = 6, scale bar = 250 μm. (**B**) Bar chart showing the percentage of Cy5^+^ neurons in different regions. (**C**) Brain tissue stained with H&E, Nissl, and Iba1^+^ immunofluorescence at 2 and 4 weeks post-drug injection. Green indicates Iba1^+^ cells. *n* = 3; scale bar = 50 μm.

Mice received intrathecal injections of the DNA origami formulations. Cervical, thoracic, and lumbar DRGs, together with sympathetic ganglia (SG), were collected 4 weeks after administration for immunofluorescence staining. The proportion of Cy5-positive neurons among total neurons was quantified under a fluorescence microscope. Results showed that neurons in cervical, thoracic, and lumbar DRG maintained a high Cy5-positivity rate (about 90%), whereas SG exhibited very low positivity (<10%), indicating strong tissue specificity of the targeted drugs (Figure 3A,B).

To evaluate potential neurotoxicity, mice received intrathecal administration of IL1RN-overexpression drugs, and brain tissues were collected at 2 and 4 weeks for HE staining, Nissl staining, and Iba1 immunofluorescence staining (Figure 3C). HE staining revealed no significant morphological abnormalities compared with the control group: neuronal somata displayed regular morphology with clear boundaries, centrally located nuclei, and no evidence of cytoplasmic eosinophilic granules; vascular walls remained intact with flattened endothelial cells and no perivascular edema or inflammatory infiltration; neuronal fiber networks were dense and homogeneous, without abnormal vacuolization or necrotic foci. Nissl staining further confirmed normal granular distribution of Nissl bodies within the cytoplasm, deeply stained nucleoli, and absence of central chromatolysis, karyopyknosis, or nuclear fragmentation; no satellite cell reactive proliferation was observed around neurons. Microglia, as the primary mediators of immune responses in brain tissue, cannot be specifically identified using the first two staining methods. To better assess neuroinflammatory states, we performed immunofluorescence staining for the macrophage marker Iba1^+^. Results showed no significant difference in the density of Iba1-positive cells compared with the control group at either 2 or 4 weeks, with most cells displaying small somata and fine, elongated processes in a resting, ramified state.

Collectively, these findings indicate that intrathecal delivery of the IL1RN-overexpression DNA origami formulation resulted in preferential accumulation in DRG neurons and did not produce obvious structural abnormalities in brain tissue at the tested dose and within the observation period.

### 3.3. Histological Evidence Suggests a Potential Role of IL1RN in Bone Defect Repair

**Figure 4:**
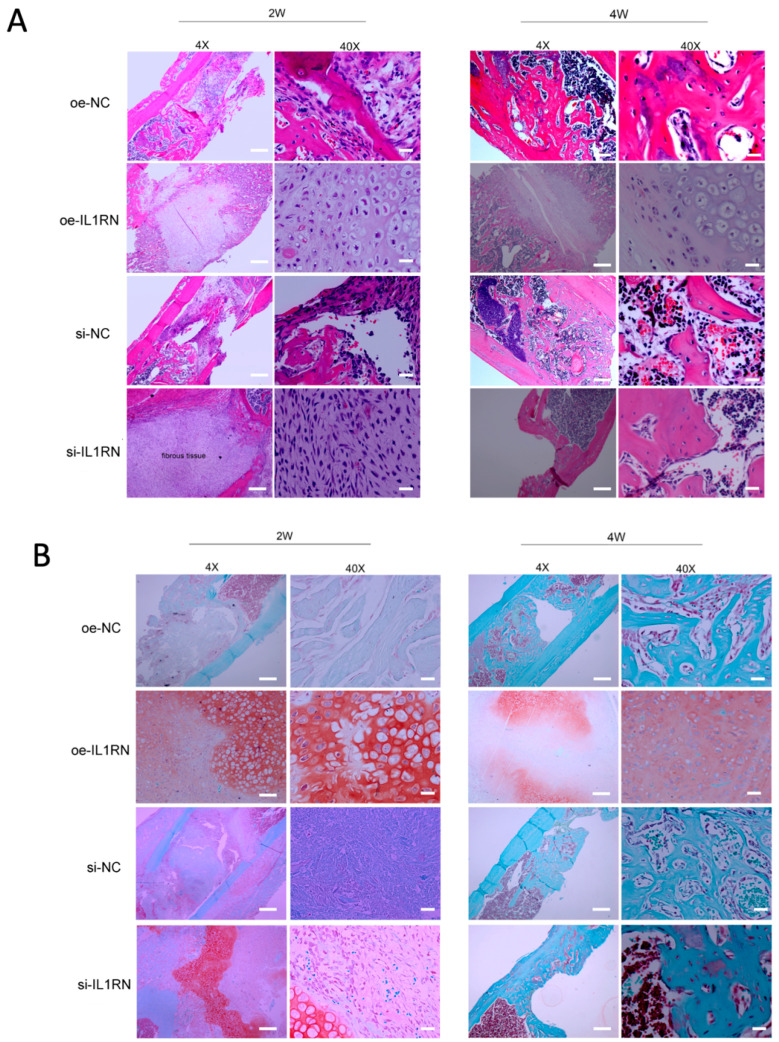
IL1RN overexpression promotes bone regeneration at bone defect sites. (**A**) Hematoxylin and eosin (HE) staining of the femur at 2 and 4 weeks post-drug injection. (**B**) Safranin-O and fast green staining of the femur at 2 and 4 weeks post-drug injection. The scale is 200 μm for 4× and 20 μm for 40×; *n* = 6.

To preliminarily explore the effect of IL1RN modulation on bone defect repair, a femoral defect model was established in mice, followed by intrathecal administration of IL1RN overexpression and knockdown drugs. Bone tissues from the defect sites were collected at 2 and 4 weeks post-treatment for histological examination (Figure 4A). Hematoxylin–eosin (H&E) staining showed that, compared with the control group, IL1RN overexpression was associated with less apparent bone resorption-related morphology at the defect site and more evident cartilage callus-like tissue during the repair phase (2 weeks), with histological features compatible with endochondral ossification at the later stage (4 weeks). In contrast, IL1RN knockdown was associated with more pronounced resorption-like changes, manifested by early fibrous tissue infiltration in the defect area, disrupted repair, and atrophic nonunion.

To further visualize the cartilage status during bone repair, Safranin O–Fast Green staining was performed (Figure 4B). The results showed that in the IL1RN overexpression group, numerous intensely red-stained cartilage islands were observed in the central region of the defect, while blue–green bone matrix deposition was detected within the peripheral cartilage, suggesting histological features consistent with endochondral ossification during the repair stage. In contrast, IL1RN knockdown was associated with deficient cartilage tissue within the defect area, with fewer apparent signs of cartilage-to-bone transformation.

### 3.4. IL1RN Modulation Is Associated with Histological Changes in the Growth Plate of the Age-Related Bone Loss Model

**Figure 5:**
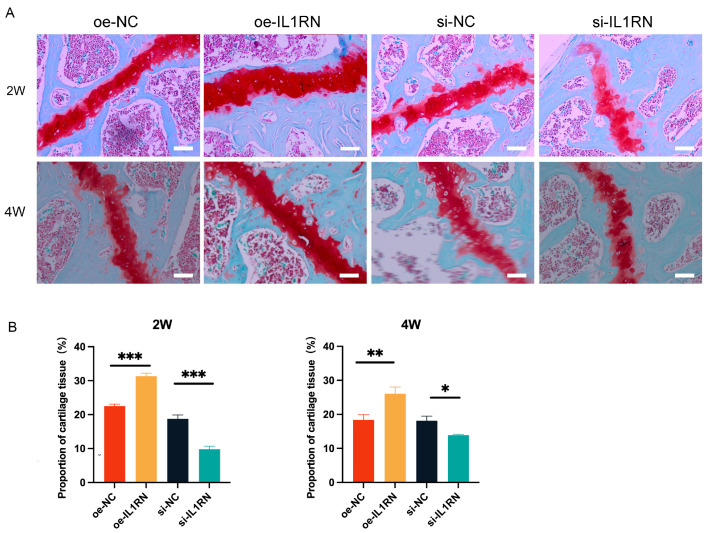
IL1RN overexpression promotes chondrocyte proliferation and protects the growth plate. (**A**) Femur stained with safranin-O and fast green. (**B**) Statistical analysis of cartilage area/total area after 2 and 4 weeks of drug injection. Scale bar= 50 μm; *n* = 6. All results are mean ± SD. * *p* < 0.05; ** *p* < 0.01; *** *p* < 0.001.

To preliminarily examine the association between IL1RN modulation and growth plate histology in the age-related bone loss model, mice received intrathecal administration of IL1RN overexpression and knockdown drugs. At 2 and 4 weeks post-treatment, the proximal femoral epiphysis was collected for analysis. Safranin O–Fast Green staining was performed, and the proportion of cartilage area relative to the total area was quantified (Figure 5A,B). Results showed that IL1RN overexpression was associated with an increased cartilage-positive area and a thicker-appearing growth plate, whereas IL1RN knockdown showed the opposite histological pattern.

## 4. Discussion

The crosstalk between the skeletal system and the nervous system is essential for maintaining bone health. As a major peripheral nerve innervating bone tissue, the upstream role of sensory nerves in bone repair has long been underestimated [22]. Following disease onset, local inflammatory stimuli within the bone microenvironment are perceived as afferent signals by receptors located on sensory nerve endings innervating bone and periosteum. This process is mediated by nociceptive nerve fibers, including Aδ myelinated fibers, which detect mechanical stimuli and transmit fast pain, and C unmyelinated fibers, which conduct slow pain while releasing neuropeptides. The specific pattern of perception depends on the expression profile of distinct nociceptive markers within neuronal subpopulations [23,24]. Meanwhile, sensory nerves undergo adaptive changes that promote axonal growth and sprouting within newly formed callus tissue via the NGF–TrkA signaling pathway [25]. Through the release of CGRP, they further enhance the transcription of osteogenic genes at the epigenetic level by modulating DNA methylation, thereby selectively guiding bone repair [26,27]. This inter-organizational crosstalk reveals that regulating sensory nerves to treat skeletal disorders represents a new therapeutic trend.

The dense structure of the bone matrix inherently restricts drug penetration. Combined with the extensive distribution of skeletal tissue, this necessitates prolonged retention of therapeutic agents within the bone and systemic circulation to facilitate their diffusion to specific lesions. Bone-targeting strategies designed to recognize general bone biomarkers typically result in non-specific distribution of drugs throughout the entire skeletal system [28]. Stimulus-responsive carriers—including enzyme-responsive, pH-responsive, and callus-responsive systems—as well as dual- or multi-targeted ligands have been developed to achieve controlled drug release at bone lesion sites [29,30]. However, the unique and dynamically changing microenvironments and molecular profiles at different repair stages make drug delivery susceptible to off-target release due to interference from exogenous signals. Effectively targeting drug delivery to specific pathological sites remains a major challenge [31]. In this study, we designed a drug delivery system targeting the dorsal root ganglion (DRG), which leverages the natural “nerve–bone axis” communication network to achieve topologically precise delivery of therapeutic payloads to bone. By exploiting the sensory nerve density gradient at injury sites, it enables spatial enrichment of drugs within bone injury zones, a process finely regulated by endogenous signaling molecules [25,32]. Therefore, delivering key neurocrine factors by targeting sensory nerves may prove more effective than directly targeting bone for drug delivery.

In this study, we designed a DNA origami carrier with a triangular three-dimensional architecture, functionalized with DRG-homing peptides to endow the drug with DRG-targeting capacity. As a natural biomacromolecule, DNA exhibits lower immunogenicity, superior biocompatibility, and biodegradability compared with traditional viral vectors or polymeric nanomaterials. Leveraging the principle of complementary base pairing, folded DNA strands can be programmably self-assembled into arbitrary predetermined structures. Significant progress has been made in DNA origami carriers for tumor-targeted therapy, gene-editing tool delivery, and precision-controlled drug release [33,34,35]. Here, we exploited the strong noncovalent interactions between streptavidin (SA) and biotin to achieve specific assembly of IL1RN proteins onto biotinylated DNA origami scaffolds [36]. By leveraging the principle of complementary base pairing, an extended chain was added to the 3′-terminus of the IL1RN siRNA fragment. This chain was then integrated with the overhanging chain extending from the DNA origami scaffold at a specific position. Ultimately, a therapeutic system capable of bidirectionally regulating IL1RN expression was constructed. Following intrathecal drug administration in mice, fluorescence tracing revealed that drug positivity rates reached 90% in sensory neurons of DRGs, while rates in sympathetic ganglia remained below 10%, confirming tissue-specific drug delivery. Western blotting further supported that the designed drug effectively modulates the expression of the IL1RN-encoded protein in DRG, consistent with its anticipated mechanism of action. Additionally, brain H&E, Nissl, and Iba1 staining did not show obvious histopathological abnormalities within the current observation period.

IL-1RN, as an endogenous inhibitor within the interleukin-1 (IL-1) family, affects nearly all cell types. Its classic function involves competitively binding to the IL-1 receptor (IL-1R1), thereby efficiently suppressing IL-1α/β-mediated inflammatory signaling. It has garnered significant attention in both inflammation and tumor research [37]. An in vitro study revealed that IL1RN promotes osteogenic differentiation induced by osteogenic culture medium in MC3T3-E1 and C3H10T1/2 cell lines. This occurs by activating the β-catenin signaling pathway through interaction with integrin β3 (ITGB3), thereby enhancing osteoblast differentiation [38]. Another study indicated that IL1RN promotes the repair of bone defects using bio-scaffolds containing rhBMP-2 (a bone morphogenetic protein) [39]. These findings suggest that IL1RN may play a positive role in bone tissue regeneration. This study investigated the role of IL1RN as a sensory neurocrine factor in regulating bone homeostasis and remodeling by administering IL1RN overexpression and knockdown drugs via intrathecal injection in mice with bone defects and osteoporosis.

Long bone injury repair involves two modes: intramembranous ossification and endochondral ossification. Compared to intramembranous ossification, which directly mediates bone formation through osteoblasts, endochondral ossification undergoes an additional phase during early healing characterized by the formation of cartilaginous callus tissue primarily driven by chondrocytes [40]. In this study, extensive cartilage islands formed in the defect area following administration of IL1RN overexpression drugs, indicating that repair proceeded via secondary healing dominated by endochondral ossification. We interpret this finding as follows: local mechanical stability is considered a key factor driving healing. When creating the bone defect model, we did not apply internal or external fixation to the femur in the defect area. Instead, we opted for simple suturing of the muscle-skin wound and did not restrict the mice’s activity throughout the entire repair process. As the primary site bearing stress during movement, the femur demands a high weight-bearing capacity. Insufficient mechanical stability in the defect area likely drove repair toward the secondary pathway of endochondral ossification [41,42,43]. Currently, differing perspectives exist regarding the mechanism underlying the transition from cartilaginous callus to bony callus. Research indicates that the Wnt/β-catenin signaling pathway plays a crucial regulatory role in directing the differentiation of cells within the cartilage-bone junction zone, promoting the transformation of chondrocytes into osteoblasts [44]. Additional studies using lineage tracing indicate that this process is mediated by reparative, postnatal skeletal stem/progenitor cells (SSPCs). SSPCs can differentiate into either chondrocytes or osteoblasts—the two skeletal lineages—enabling fracture healing to transition from the cartilage-healing phase to the bone-healing phase [45]. Furthermore, our findings suggest that IL1RN itself possesses the potential to promote cartilage tissue regeneration in defect areas. A study investigating the role of engineered macrophages in self-regulating IL1RN expression in response to IL-1β-mediated inflammatory responses indicated that upregulation of IL1RN expression protects the cartilage matrix (sulfated glycosaminoglycans, sGAGs) from degradation [46]. These findings further support the hypothesis that IL1RN possesses both protective and proliferative potential in chondrogenesis.

Following intrathecal injection of IL1RN knockdown drugs, reduced local secretion of anti-inflammatory mediators prevented timely resolution of the inflammatory phase at the injury site. Bone tissue section staining revealed fibrous tissue occupying the defect area, with subsequent nonunion developing in later stages. We hypothesize that excessively activated IL-1 at the injury site drives osteoclast differentiation by activating the RANK signaling pathway [47]. Excessive bone resorption is a key factor in repair failure. Hematoxylin and eosin staining revealed sparse erythrocytes in the inter-trabecular marrow spaces of the IL1RN knockdown group, suggesting potential circulatory disorders, which may represent another contributing factor to repair failure.

Single nucleotide polymorphisms in IL1R1 and IL1RN between healthy individuals and osteoporosis (OP) patients were revealed to be associated with genetic susceptibility to OP [48]. In this study, IL1RN promotes increased thickness of the femoral growth plate in the age-related bone loss model. This phenomenon may be related to IL-1 activating transcription factors NFATc1/cFos through synergistic activation of IL-1R/RANK receptors, thereby upregulating IL-1R expression and initiating the osteoclast differentiation process [49]. IL1RN can inhibit osteoclast proliferation by competitively binding to IL-1R, thereby mitigating bone resorption in osteoporosis. In osteoarthritis, inflammatory mediators such as IL-1 stimulate nitric oxide (NO) production via the inducible nitric oxide synthase (iNOS) pathway, activating matrix metalloproteinases (MMPs) to degrade the extracellular matrix of cartilage, impairing chondrocyte metabolism and inducing apoptosis. Current evidence further indicates that the Hippo/YAP signaling pathway in chondrocytes participates in regulating the aforementioned pathological changes [50,51]. Based on the results of this study, we speculate that IL1RN may protect the epiphyseal growth plate in age-related bone loss models through the aforementioned mechanisms.

This study also has the following limitations: Although the present findings indicate that DRG IL1RN modulation affects bone repair in vivo, the direct effects of sensory neuron-derived IL1RN on osteoblasts, osteoclasts, and chondrocytes remain to be clarified. In addition, the evaluation of bone repair in the present study was mainly based on histological observations, and more quantitative analyses, such as micro-CT-based assessment of new bone formation and defect repair, should be performed. Further investigations using in vitro cell models, neuron–skeletal cell co-culture systems, quantitative bone repair analyses, and pathway-specific approaches are needed to define the downstream cellular and molecular mechanisms more comprehensively. Although agarose gel electrophoresis and AFM were used to characterize formulation assembly, the pharmaceutical characterization remains preliminary, particularly with respect to stability under biologically relevant conditions. Systematic longitudinal studies are needed to determine whether structural integrity and cargo retention can be maintained over time. The neurotoxicity assessment was also limited and should be regarded as preliminary. Although no obvious brain histopathological abnormalities were observed within the current observation period, more comprehensive tissue-level, functional, and long-term safety evaluations are required before drawing firm conclusions regarding the safety of repeated intrathecal administration.

## 5. Conclusions

In summary, through the design of a drug delivery system targeting dorsal root ganglia using DNA origami as a carrier and the sensory neurocrine factor IL1RN as the active pharmaceutical ingredient, preliminary exploration in bone defect and age-related bone loss models revealed that IL1RN inhibits bone resorption at defect sites while promoting bone regeneration. Knockdown of IL1RN, conversely, enhances bone resorption at defect sites while inhibiting new bone regeneration, leading to prolonged non-healing of bone defects. Furthermore, IL1RN protects the growth plate in age-related bone loss models. These findings suggest that IL1RN may function as a crucial sensory nerve-derived neurocrine factor involved in bone remodeling and metabolism. However, the downstream cellular targets and molecular mechanisms underlying these observations remain incompletely defined. Future studies are needed to determine how sensory neuron-derived IL1RN regulates osteoblasts, osteoclasts, and chondrocytes, and the related signaling pathways, during bone remodeling and repair.

## Data Availability

The original contributions presented in this study are included in the article’s Supplementary Materials. Further inquiries can be directed to the corresponding author.

## Supplementary Materials

The following supporting information can be downloaded at https://www.mdpi.com/article/10.3390/pharmaceutics18070898/s1. Supplementary File S1 (oligonucleotide sequences for the oe-IL1RN formulation): Table S1, Sequences of biotinylated oligonucleotides used in this work; Table S2, Sequences of peptide-capture oligonucleotides used in this work; Table S3, Sequences of 5′-Cy5-modified oligonucleotides used in this work; Table S4, Sequences of other staple oligonucleotides used in this work; Table S5, Sequence of the linker oligonucleotide used in this work. Supplementary File S2 (oligonucleotide sequences for the si-IL1RN formulation): Table S6, Sequences of siRNA-capture oligonucleotides used in this work; Table S7, Sequences of peptide-capture oligonucleotides used in this work; Table S8, Sequences of 5′-Cy5-modified oligonucleotides used in this work; Table S9, Sequences of other staple oligonucleotides used in this work; Table S10, Sequence of the linker oligonucleotide used in this work.
